# Racial and Ethnic Disparities in Surgical Timing and Preoperative Care for Urinary Incontinence: a Large Single Academic Institution Study in the USA

**DOI:** 10.1007/s00192-025-06451-0

**Published:** 2026-01-30

**Authors:** Julia Shen, Rachan Ghandour, Maria Micussi, Angela Marses, Susanna Ovsepian, Knar Krafian, Sasha Aljamal, Vatche A. Minassian

**Affiliations:** 1 Division of Urogynecology, Department of OB/GYN, Brigham and Women’s Hospital, Boston, MA USA; 2 University of Michigan Medical School, Ann Arbor, MI USA; 3 Physiotherapy Department, Federal University of Rio Grande Do Norte Center, Natal, Brazil

**Keywords:** Health disparities, Surgery, Urinary Incontinence

## Abstract

**Introduction and Hypothesis:**

This retrospective cohort study examined racial/ethnic differences in time from initial presentation with urinary incontinence (UI) to surgery and number of clinical visits prior to sling surgery for UI.

**Methods:**

Electronic health records were accessed for patients aged 18 years or older who underwent sling procedures for UI over a 134-month period at a single academic institution. The primary outcome was lead time to surgery (LTTS), the number of days between the first clinical visit for UI and the anti-incontinence surgery. The secondary outcome was the number of UI-related clinical visits prior to surgery. ANOVA and multivariable linear regression analyses were used to identify independent predictors.

**Results:**

A total of 4246 patients were analyzed. On univariate analysis, Black patients had the longest LTTS (462.8 ± 735.7 days) compared with white patients (302.2 ± 496.8 days) and “Other” patients (226.4 ± 429.4 days; *p* < 0.01). However, multivariable regression revealed that racial/ethnic differences in LTTS were not statistically significant after controlling for confounders (β = 11.13, *p* = 0.41). Instead, age and UI severity were identified as significant predictors of LTTS. Age was inversely associated with LTTS (β = −2.53, *p* < 0.02), and greater UI severity was associated with shorter LTTS (β = −21.93, *p* = 0.03). Notably, racial/ethnic differences in preoperative visit frequency remained significant in adjusted models; Asian and Hispanic patients had, on average, 0.90 (*p* < 0.01) and 0.58 (*p* < 0.01) more visits respectively than white patients.

**Conclusions:**

Although lead time to UI surgery did not differ by race/ethnicity after adjustment, Asian and Hispanic patients had significantly more preoperative visits prior to surgery.

## Introduction

Urinary incontinence (UI) is a common chronic condition, affecting an estimated 38–62% of the female population and profoundly impacting quality of life [1–3]. Despite its high prevalence, only 1 in 10 American women undergo surgical treatment for UI, with most managing symptoms through costly alternatives such as absorbent products, medications, and other conservative therapies [3]. Identifying what factors delay or limit access to timely surgical care for UI is therefore essential to improving equity in treatment delivery.

A small but growing body of research has documented racial and ethnic disparities in UI surgery. Previous studies have found that Black and Hispanic women are less likely to undergo mid-urethral sling procedures than their white counterparts [4]. Postoperative complications and adverse events have also been shown to be more prevalent among non-white women [3–5]. However, these studies have primarily focused on whether patients receive surgical treatment and what happens after surgery, leaving critical gaps in our understanding of the care processes between initial clinical presentation and surgical intervention [3–9].

Furthermore, although existing research has documented disparities in surgical utilization rates, surgical approaches, and postoperative outcomes, little is known about the temporal aspects of UI care delivery—specifically, no data exist on preoperative visit burden or lead time prior to sling surgery for UI [10]. These temporal patterns warrant investigation because delays in care progression may reflect systemic barriers, differences in provider decision making, or patient-level factors that have the potential to be addressed through targeted interventions.

In this retrospective cohort study, we investigated whether there are racial and ethnic differences in the time from patients’ first clinical presentation with UI to their date of surgery for UI. Our secondary objective was to examine whether there are racial and ethnic differences in the number of clinical visits patients have for UI before receiving surgical treatment for UI. We hypothesized that non-white patients experience longer waiting periods and require more clinical visits before receiving surgical treatment than white patients. By examining process-level measures rather than surgical outcomes alone, this study provides foundational evidence for disparities in care progression and establishes the groundwork for future efforts to improve timely, equitable access to UI treatment.

## Materials and Methods

A retrospective cohort study was conducted using de-identified electronic health records from the Research Patient Data Registry (RPDR) and the patient electronic health records (EHRs) database at a tertiary care center in Boston, Massachusetts, USA. Patients aged 18 years and older who underwent sling procedures for UI between 1 January 2013, and 13 March 2024 were included. The cohort was identified by first using the International Classification of Diseases (ICD)−10 codes for stress or mixed UI, followed by the Current Procedural Terminology (CPT)−4 code [57288] for anti-incontinence sling operations for stress or mixed UI (fascia or synthetic) within the specified dates.

Participants were included if they were self-reported female sex, over 18 years of age, and identified as having undergone a sling operation for UI based on ICD-10 and CPT-4 codes. Race and ethnicity were self-reported and categorized as white, Black, Hispanic, Asian, and “Other.” For clarity in reporting, we use the following terminology: “non-white,” referring to participants who do not identify as white (including Hispanic, Black/African American, Asian, American Indian/Alaska Native, Pacific Islander, and Native Hawaiian); “Black” refers to Black/African American participants; “Other” includes all participants who are American Indian/Alaska Native, Pacific Islander, Native Hawaiian, or those listed as mixed race (more than one race listed). For analytic purposes, we combined racial/ethnic groups with fewer than 10 patients into an “Other” category (American Indian/Alaska Native, Pacific Islander, Native Hawaiian, or those with more than once race listed). This approach was chosen to preserve statistical power, reduce model instability, and protect patient confidentiality, but we acknowledge that collapsing these groups may mask meaningful differences. Those listed as being of Hispanic ethnicity, regardless of listed race, are considered Hispanic; those listed as being of non-Hispanic ethnicity in the EHRs database are listed as their given race.

Exclusion criteria included patients with unknown race or ethnicity in their EHR, and those who had undergone multiple or previous procedures for UI prior to the specified date range. Patient sociodemographic information and other variables including age, race, body mass index (BMI), language, gravidity, parity, insurance type, employment status, sexual activity, duration of UI symptoms, UI type, UI severity, surgery location, and provider specialty were collected. UI severity was calculated based upon patient questionnaire responses for the Sandvik Severity Index, a well-validated scale that utilizes the reported frequency of UI and the reported amount of leakage to classify UI as “continent,” “mild incontinence,” “moderate incontinence,” “severe incontinence,” and “very severe incontinence” [11].

Our primary aim was to determine racial and ethnic differences in the lead time to sling procedures for UI. Lead time to surgery (LTTS) was defined as the duration of time (days) between a patient's first clinical visit reporting UI and their sling procedure date. Our secondary outcome of interest included the assessment of sociodemographic differences in the number of clinical visits between the patient’s first clinical visit for UI and the patient’s ultimate sling surgery for UI.

All statistical data were analyzed using RStudio version 2022.07.2 + 576, and data visualization was completed using GraphPad Prism version 9.0.0. Data were presented as mean ± standard deviation (SD) or median and interquartile range (IQR) for continuous variables and as absolute frequencies and percentages for categorical variables. We compared demographic and clinical characteristics between racial groups using one-way ANOVA and Chi-squared tests for continuous and categorical variables respectively. Multivariable linear regression was performed, and variables were included in the model if they yielded *p* < 0.05 on univariate analysis, improved the adjusted R^2^, and/or were identified as confounding factors. Values of *p* < 0.05 were considered to indicate statistical significance.

To determine the study sample size, we first conducted a preliminary study analysis on a random sample of 100 patients of the three most represented racial/ethnic groups at our institution including white, Black, and Hispanic patients; we calculated the mean and SD for the LTTS in each group. Based on these data, we estimated an effect size of 1.59. Then, using a significance level of 0.05 and a power of 0.80, we determined that a minimum of 128 patients would be needed for each group.

## Results

**Fig. 1:**
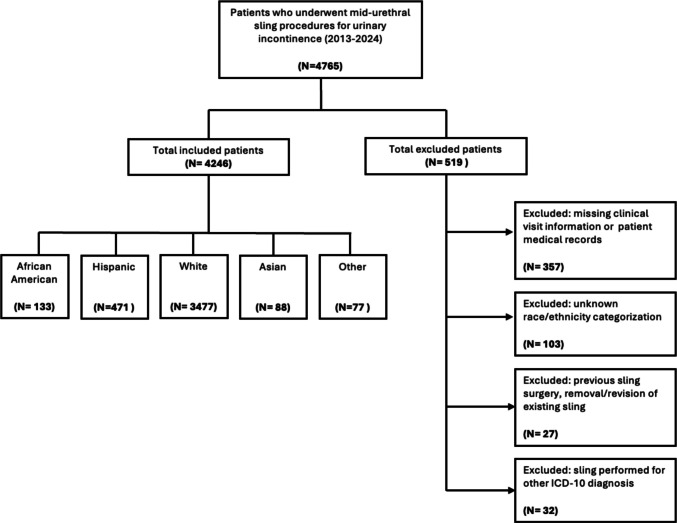
Participant flow diagram. *ICD* International Classification of Diseases

A total of 4765 patients who underwent sling placement for SUI or MUI were identified from the RPDR database. After excluding patients with unknown race/ethnicity (*n* = 103), missing clinical visit information (*n* = 357), previous sling surgery (*n* = 27), and procedures performed for diagnoses other than UI (*n* = 32), our final cohort comprised 4246 patients (Fig. 1). The study cohort consisted of 81.9% white, 11.1% Hispanic, 3.1% Black, 2.1% Asian, and 1.8% “Other” patients.

Significant differences emerged across racial/ethnic groups for several characteristics (*p* < 0.05). White women (age = 56.4 years) were older at presentation than Black women (age = 51.4 years) or Hispanic women (age 49.4 years), *p* < 0.05. Black women tended to have higher BMIs (BMI = 31.5 kg/m^2^) whereas Asian women (BMI = 25.5 kg/m^2^) had lower BMIs on average, *p* < 0.05. Employment rates were highest among white patients (51.6%), and the lowest in Hispanic (45.2%) and “Other” patients (37.7%), *p* < 0.05. Private insurance was the most common form of coverage across all groups, ranging from 43.1% (Hispanic) to 59.1% (Asian). Other factors significantly associated with race/ethnicity included gravidity and parity (Table 1).

**Table 1:** Sociodemographic characteristics according to race/ethnicity (*N* = 4246)

Variables	White (*n* = 3477)	Black (*n* = 133)	Hispanic (*n* = 471)	Asian (*n* = 88)	Other (*n* = 77)	*p* value
Age, years, mean ± SD	56.4 ± 11.7^a,b^	51.4 ± 11.5^a^	49.4 ± 11.5^b,c^	53.4 ± 12.6^c^	52.7 ± 12.2	* < 0.05*
BMI, kg/cm^2^, mean ± SD	27.7 ± 5.8^a,b,c,d^	31.5 ± 6.8^a,e,f^	29.7 ± 5.1^b,e,g^	25.5 ± 3.9^c,f,g,h^	30.6 ± 5.9^h^	* < 0.05*
Employment, *n* (%)						* < 0.05*
Employed	1795 (51.6)	66 (49.6)	213 (45.2)	41 (46.6)	29 (37.7)	
Retired	431 (12.4)	4 (3.0)	19 (4.0)	9 (10.2)	2 (2.6)	
Unemployed	355 (9.6)	19 (14.3)	101 (21.4)	13 (14.8)	12 (15.6)	
Disability	41 (1.2)	2 (1.5)	10 (2.2)	1 (1.1)	1 (1.3)	
Unknown	875 (25.2)	42 (31.6)	128 (27.2)	24 (27.3)	33 (42.8)	
Insurance, *n* (%)						* < 0.05*
Private	1778 (51.1)	68 (51.1)	203 (43.1)	52 (59.1)	38 (49.4)	
HMO	189 (5.4)	10 (7.5)	28 (5.9)	4 (4.5)	2 (2.6)	
Medicare	1215 (34.9)	24 (18.0)	88 (18.7)	20 (22.7)	22 (28.6)	
Medicaid/MassHealth	152 (4.4)	15 (11.3)	109 (23.1)	4 (4.5)	8 (10.3)	
Self-pay	79 (2.3)	5 (3.8)	18 (3.8)	3 (3.5)	0 (0.0)	
Other	43 (1.3)	9 (6.8)	19 (4.0)	5 (5.7)	7 (9.1)	
Unknown	21 (0.6)	2 (1.5)	6 (1.4)	0 (0.0)	0 (0.0)	
Gravidity, mean ± SD	2.9 ± 1.6^a,b^	4.1 ± 2.1^a,c,d,e^	3.6 ± 2.4^b,c,f^	3.0 ± 1.8^d,f^	3.4 ± 1.7^e^	* < 0.05*
Parity, mean ± SD	2.3 ± 1.2^a,b^	2.9 ± 1.5^a,c^	3.0 ± 1.5^b,d^	2.4 ± 1.1^c,d^	2.7 ± 1.2	* < 0.05*

The continuous variables are expressed as mean and standard deviation and the categorical data are expressed as relative frequency (%) and absolute frequency (*n*)

Not all patients had a response recorded to every single question

*BMI* body mass index, *HMO* Health Maintenance Organization, *SD* standard deviation

Data in italics indicate a significant difference (*p* < 0.05)

The Chi-squared test was applied to categorical variables and one-way ANOVA to continuous variables

Identical letters (^a^, ^b^, ^c^, ^d^, ^e^, ^f^, ^g^, ^h^) between the groups indicate the difference between the times according to Tukey post-hoc

**Fig. 2:**
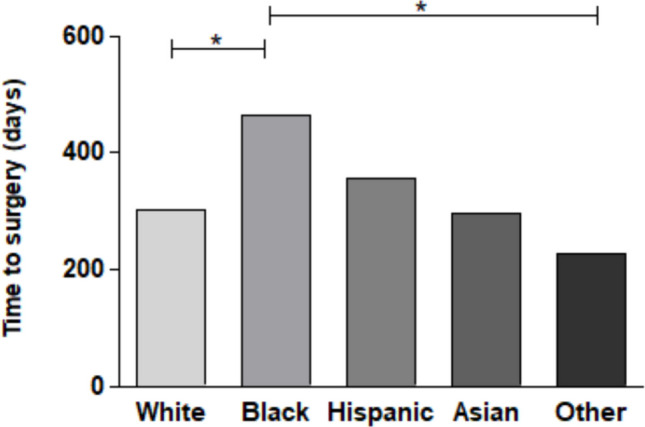
Mean time to surgery according to race. *Significant in the one-way ANOVA test

Univariate analysis revealed that Black patients had the longest mean LTTS (462.8 ± 735.7 days) compared with Hispanic (355.0 ± 545.7), white (302.2 ± 496.8 days), Asian (296.4 ± 432.0), and Other patients (226.4 ± 429.4 days; *p* < 0.01; Fig. 2). Black patients also had the longest median LTTS (154.0 [69.0–530.0]) compared with Hispanic (146.0 [62.5–377.0]), Asian (134.5 [63.5–262.8]), white (127.0 [65.0–296.0]), and Other patients (102.0 [56.5–178.0]). Severity of UI varied significantly across groups (*p* < 0.05), with “severe” symptoms being most common among all racial groups (28.0–44.4%). Most patients underwent concomitant procedures with their sling surgery (61.0–69.2%), although this did not differ significantly by race (*p* = 0.77). Treatment was predominantly provided by urogynecologists (48.9–65.9%), with significant variation in specialty distribution across racial groups (*p* < 0.05; Table 2).

**Table 2:** Information on symptoms and treatment of urinary incontinence by race/ethnicity (*N* = 4246)

Variables	White (*n* = 3477)	Black (*n* = 133)	Hispanic (*n* = 471)	Asian (*n* = 88)	Other (*n* = 77)	*p* value
Time to surgery (days)						
Mean ± SD	302.2 ± 496.8^a^	462.8 ± 735.7^a,b^	355.0 ± 545.7	296.4 ± 432.0	226.4 ± 429.4^b^	* < 0.01*
Median (IQR)	127.0 (65.0—296.0)	154.0 (69.0—530.0)	146.0 (62.5—377.0)	134.5 (63.5—262.8)	102.0 (56.5—178.0)	
Numbers of visits						
Mean ± SD	3.6 ± 2.3	3.8 ± 2.0	3.9 ± 2.4	3.8 ± 2.2	3.3 ± 2.3	0.16
Median (IQR)	3.0 (2.0—4.0)	3.0 (2.0—5.0)	3.0 (2.0—5.0)	3.5 (2.0—4.0)	3.0 (2.0—4.0)	
Symptom duration (months), mean (SD)	38.3 ± 69.4	36.1 ± 50.6	44.3 ± 85.9	41.4 ± 67.2	52.9 ± 66.2	0.07
Severity, *n* (%)						* < 0.05*
Continent	312 (14.4)	15 (20.0)	15 (6.0)	6 (13.0)	7 (15.6)	
Mild	345 (15.9)	9 (12.0)	31 (12.3)	9 (19.6)	8 (17.8)	
Moderate	544 (25.1)	20 (26.7)	67 (26.7)	10 (21.7)	5 (11.1)	
Severe	717 (33.2)	21 (28.0)	87(34.7)	14 (30.4)	20 (44.4)	
Very severe	248 (11.4)	10 (13.3)	51 (20.3)	7 (15.3)	5 (11.1)	
Prior treatment, *n* (%)^c^						0.98
No	1491 (42.1)	61 (45.5)	202 (42.0)	39 (43.4)	38 (49.4)	
Surgery	136 (3.8)	3 (2.3)	19 (3.9)	4 (4.3)	1 (1.2)	
Conservative	1918 (54.1)	70 (52.2)	260 (54.1)	47 (52.3)	38 (49.4)	
Concomitant surgery, *n* (%)						0.77
Yes	2335 (67.2)	92 (69.2)	300 (63.7)	56 (63.6)	47 (61.0)	
No (only sling)	1142 (32.8)	41 (30.8)	171 (36.3)	32 (36.4)	30 (39.0)	
Surgeon's specialty, *n* (%)						* < 0.05*
Urogynecology	2198 (63.2)	65 (48.9)	290 (61.6)	58 (65.9)	42 (54.5)	
Female urology	95 (2.7)	6 (4.5)	14 (3.0)	4 (4.6)	9 (11.7)	
General gynecology	976 (28.1)	45 (33.8)	114 (24.2)	20 (22.7)	22 (28.6)	
General urology	191 (5.5)	16 (12.0)	51 (10.8)	6 (6.8)	4 (5.2)	
Other	17 (0.5)	1 (0.8)	2 (0.4)	0 (0.0)	0 (0.0)	

The continuous variables are expressed as mean and standard deviation, and the categorical data are expressed as relative frequency (%) and absolute frequency (*n*)

Not all patients had a response recorded to every single question

*SD* standard deviation, *IQR* interquartile range

Data in italics indicate a significant difference (*p* < 0.05)

The Chi-squared test was applied to categorical variables and one-way ANOVA to continuous variables

Identical letters (^a^, ^b^) between the groups indicate the difference between the times according to Tukey post-hoc

^c^Some patients underwent surgery and conservative treatment

Multivariable regression analysis of time to surgery identified only age (β = −2.53, *p* < 0.02) and UI severity (β = −21.93, *p* = 0.03) as significant predictors (Table 3). Although initial univariate analysis showed significant racial/ethnic differences in surgical timing, these disparities were no longer significant after controlling for confounding variables in the regression model (β = 11.13, *p* = 0.41). Instead, age at presentation and UI severity emerged as the primary drivers of surgical timing. For each 1-year increase in age, time to surgery decreased by approximately 2.5 days, whereas increasing UI severity was associated with a 22-day reduction in time to surgery. Other factors, including BMI (β = −1.29, *p* = 0.54) and insurance status (β = −5.85, *p* = 0.52), did not significantly impact surgical timing.

**Table 3:** Linear regression analysis estimating the effect of race on lead time to UI surgery controlling for sociodemographic and clinical variables

	β-coefficient	Standard error	*p* value	95% confidence interval
Age	−2.53	1.09	* < 0.02*	−4.68; −0.38
BMI	−1.29	2.12	0.54	−5.46; 2.88
Race	11.13	13.63	0.41	−15.67; 37.93
Employment	5.12	7.49	0.49	−9.61; 19.85
Insurance	−5.85	9.16	0.52	−24.00; 12.30
Parity	−9.49	9.46	0.32	−28.04; 9.06
Severity of UI	−21.93	10.03	*0.03*	−41.64; −2.22
Duration of symptoms	−0.17	0.17	0.33	−0.50; 0.16
Surgeon's Specialty	−55.4	64.30	0.39	−181.56; 70.76

*BMI* body mass index, *UI* urinary incontinence

Data in italics indicate a significant difference (*p* < 0.05)

A separate multivariable regression analysis examining factors associated with the number of preoperative visits identified three significant predictors: age (β = 0.02, *p* < 0.001), race (β = 0.20, *p* < 0.01), and surgeon's specialty (β = −0.60, *p* = 0.03; Table 4). Confounding variables adjusted for in our analysis included age, BMI, race, employment, insurance, parity, severity of UI, duration of symptoms, and surgeon’s specialty. Race played a significant role, with Asian patients averaging 0.90 more visits and Hispanic patients having 0.58 more visits than white patients (*p* < 0.01; Table 4). Older patients also tended to have slightly more preoperative visits, with each additional year of age corresponding to an increase of 0.02 visits. Additionally, surgeon specialty influenced visit frequency, as patients treated by general urologists required fewer preoperative visits than those seeing urogynecologists (β = −0.601, *p* = 0.03). Other variables were not found to significantly impact the number of preoperative visits (Table 4). We calculated variance-inflation factors (VIFs) for all confounding variables and found no evidence of major collinearity (all VIFs < 1.3).

**Table 4:** Linear regression analysis estimating the effect of race on number of UI clinical visits prior to surgery controlling for sociodemographic and clinical variables

	β-coefficient	Standard error	*p* value	95% confidence interval
Age	0.024	0.005	* < 0.001*	0.01; 0.03
BMI	−0.006	0.009	0.54	−0.02; 0.01
Race (white = reference)				
Asian	0.904	0.394	*0.02*	0.13; 1.68
Hispanic	0.575	0.177	* < 0.01*	0.23; 0.92
Employment	0.029	0.033	0.37	−0.04; 0.10
Insurance	0.003	0.041	0.93	−0.08; 0.08
Parity	−0.040	0.042	0.34	−0.12; 0.04
Severity of UI	−0.027	0.044	0.53	−0.11; 0.06
Duration of symptoms	−0.001	0.001	0.09	−0.003; 0.001
Surgeon's specialty (urogynecology = reference)				
General urology	−0.601	0.284	*0.03*	−1.16; −0.04

*BMI* body mass index, *UI* urinary incontinence

Data in italics indicate a significant difference (*p* < 0.05)

## Discussion

For patients diagnosed with SUI or MUI within our integrated health care system, no significant racial or ethnic differences in lead time to sling surgery were observed after controlling for health care provider and patient-level confounders. Age and severity of UI symptoms were the key drivers of shortened LTTS. However, notable racial and ethnic disparities were observed in preoperative visit burden: Asian and Hispanic patients required 0.90 and 0.58 more visits respectively than their white counterparts before receiving surgical care. This suggests that although lead time to surgery was similar across racial groups, Asian and Hispanic patients often still face a more complex preoperative process.

There is very limited research examining racial and ethnic disparities in urogynecological surgery, but existing studies suggest that non-white women are less likely to receive surgical treatment for UI. One study found that Black and Asian women were less likely to be offered surgical options for UI, often requiring multiple consultations before receiving definitive treatment recommendations [6]. Similarly, another study demonstrated that Spanish-speaking women were less likely to be offered surgery for POP than English-speaking patients [12]. In a separate analysis, women from a racial minority were found to have had a 20–50% lower odds of undergoing sling procedures and a 40–100% increased likelihood of receiving conservative management [13]. Taken together, these studies suggest that non-white women might face greater barriers to obtaining surgical treatment for UI, which is consistent with our observation that Asian and Hispanic patients experienced a higher preoperative visit burden prior to surgery.

It is important to note that our findings should be interpreted within the context of current treatment guidelines for UI: before surgical intervention is offered, patients are generally counseled on conservative therapies for incontinence (i.e., pelvic-floor physiotherapy, anticholinergic medications). Surgery is not typically offered at the initial consultation [14]. Given the design and scope of this study, we are unable to determine whether a longer LTTS or greater preoperative visit burden stems from patient-level factors (e.g., hesitancy toward surgery, preference for conservative management), structural-level factors (e.g., provider bias, transportation barriers), or some combination of the above. In effect, it is likely that several interconnected factors drive the disparities in preoperative visit burden that we observed.

For example, communication challenges, particularly across cultural and linguistic differences, may necessitate additional encounters [15]. Provider bias—whether implicit or explicit—might lead to delays in surgical management for certain patients [16]. Limited access to subspecialty care within communities can result in fragmented referral patterns [17]. Additionally, the higher burden of medical comorbidities sometimes seen in underrepresented populations may require extra preoperative clearance steps; previous studies have demonstrated that patients with a greater number of co-morbidities often face longer surgical lead times [17, 18]. The culmination of these challenges in navigating the health care system can result in repeated consultations and delays in receiving surgical treatment. Aside from structural barriers, differing cultural perceptions surrounding UI and its treatment could also potentially play a role in hesitancy in undergoing surgery, leading to more attempts at conservative management or visits before surgery [19, 20]. For instance, Siddiqui et al. reported that although both white and Black women expressed the belief that treatment options existed for UI, this belief was not expressed in Latina focus groups. Latina women endorsed maintaining more secrecy about UI than other women and reported longer delays in seeking care [21].

Our finding that racial differences in surgical timing were insignificant after adjustment for confounders parallels the results from another study in an integrated health care system [22]. Research on POP surgery within a managed care system similarly found no racial, ethnic, or socioeconomic disparities in surgical approach after accounting for patient factors, such as prolapse stage, patient comorbidities, sociodemographic factors, and medical center. Deandrade et al. suggest that structural-level factors within health care systems—including referral patterns, care coordination, and access to healthcare—might be more influential in driving observed disparities [6].

After controlling for sociodemographic factors and clinical characteristics in our analysis, age at presentation and severity of UI symptoms emerged as the most significant drivers of lead time to surgery. The inverse relationship between age and surgical waiting time likely reflects clinical decision-making priorities. Older patients may demonstrate a greater willingness to pursue surgical intervention owing to a longer duration with symptoms, more significant impact on quality of life or more flexibility in their schedules, whereas younger women may encounter challenges in arranging time off work for surgery. Correspondingly, the association between increased UI severity and shorter time to surgery suggests appropriate clinical prioritization, with providers expediting care for the most severely affected patients regardless of race or ethnicity.

The observation that Asian and Hispanic patients face a higher preoperative visit burden highlights potential inefficiencies in care delivery that may exacerbate disparities in health care access and satisfaction. Additional visits translate into more time off work, higher transportation costs, and increased exposure to potential insurance barriers, particularly for populations already at greater risk for financial strain. Future efforts to streamline preoperative care by standardizing surgical counseling protocols and reducing redundant consultations could mitigate these inequities. Furthermore, understanding whether these disparities are associated with worse patient-centered outcomes—such as higher dropout rates or lower patient satisfaction—is an important next step.

Our study had some weaknesses. Our data depended on the reliability of the ICD-10 diagnosis and the corresponding CPT-4 codes for sling surgeries. We do not have information on other surgeries that patients may have undergone, including retropubic urethropexies or urethral bulking agents, and whether those are differentially offered to patients of different races or ethnicities; however, as mid-urethral slings are considered the gold standard for SUI management, we believe that our findings are a valuable addition to the literature. A possible limitation to our study is that although our finding that Asian and Hispanic patients have 0.90 and 0.58 more preoperative visits respectively was statistically significant, the effect size is small. As such, the clinical relevance of this is more nuanced: for some patients, an additional visit may represent minimal disruption, but for others, even modest differences in visit frequency may contribute to increased patient inconvenience, financial burden, and opportunity costs, particularly among working-age patients or those with limited access to transportation and childcare. Another limitation is that, given its retrospective nature, we were limited in our ability to comment on causality between race/ethnicity and lead time to surgery, or the number of clinical visits prior to surgery. The retrospective study design also introduces the possibility of misclassification and selection bias; to limit these factors, we employed clear and detailed inclusion/exclusion criteria within our database. Our patients’ EHRs lacked information on sociodemographic variables such as income and education level that may influence LTTS and preoperative visit frequency. To account for these factors as far as possible, we instead used available data on employment status and insurance type as proxies for patients’ socioeconomic level. Although the RPDR includes data for different clinical sites across the state of Massachusetts, our sampling from one single institution limits the generalizability of this study. However, our study sample included a sizable number of non-white women and we did achieve the required power for significance in Black and Hispanic women. Owing to the smaller numbers of Native American/American Indian and Pacific Islander patients available in RPDR, the conclusions that we can draw for these populations are limited. The global generalizability of our findings is limited as well, given the differences between US health care infrastructure/insurance systems and those of other countries.

Conversely, our use of the RPDR together with patient EHRs also provided several methodological strengths to this study. The extensive EHR database in our health care system allowed for uninterrupted observability of the longitudinal care timelines of > 4000 patients, granting access to exact dates of initial presentation, subsequent visits, and surgery as an endpoint. Furthermore, the availability of detailed data for all health care encounters uniquely enabled us to assess UI severity and symptom duration as variables. By evaluating both surgical timing and preoperative visit frequency in our study, we were able to examine temporal aspects of care quality rather than focusing solely on final treatment provision; even for patients who ultimately received appropriate surgical treatment, the path to that treatment may be more complex or more delayed for some. To our knowledge, this is a unique study that assesses disparities in preoperative visit frequency and lead time to surgical treatment of UI. It offers a different perspective of how the care processes of patients from racial–ethnic minorities may be differentially affected.

To conclude, although no significant racial or ethnic disparities in lead time to UI surgery were observed after accounting for clinical and patient-level confounders, we found that Asian and Hispanic patients required significantly more preoperative visits than white patients before receiving surgical treatment. These findings suggest that there might be persistent racial differences in preoperative care pathways for UI, despite equivalent LTTS outcomes. Future studies are needed to examine the specific factors contributing to increased visit burden, evaluate interventions to streamline the UI surgical care pathway, and incorporate measures, such as the time from surgery being offered to surgery being performed, to better understand preoperative variation.

## Data Availability

This study used third-party data made available under license that the author does not have permission to share. Requests to access the data should be directed to Dr. Vatche Minassian at vminassian@bwh.harvard.edu.
